# Maternal Determinants of Retinopathy of Prematurity (ROP): Spotlight on Gestational Diabetes Mellitus

**DOI:** 10.7759/cureus.105424

**Published:** 2026-03-18

**Authors:** Syed Abdur Rahman Farzan, Mohammed Abdul Saleem, Shifa Zaidi, Maktha Vijay, Mirza Amer Ali Baig

**Affiliations:** 1 Pediatrics, Gandhi Hospital, Hyderabad, IND; 2 Community and Preventive Medicine, Gandhi Hospital, Hyderabad, IND

**Keywords:** gestational diabetes mellitus (gdm), low birth weight, neonatal risk factors., preterm infants, retinopathy of prematurity

## Abstract

Background: Retinopathy of Prematurity (ROP) is increasingly recognized as one of the major causes of preventable blindness in India and other low- and middle-income countries, particularly affecting preterm infants. Identifying risk factors is crucial for early detection and prevention. Our study aims to reevaluate determinants of ROP among preterm and low birth weight neonates admitted to a tertiary care center in South India.

Methodology: A retrospective observational study was conducted in the neonatal intensive care unit of a tertiary care hospital, including preterm neonates with gestational age <37 weeks and/or birth weight <2500 g. The total sample size was 100, and the study was conducted over three months. Univariate analysis was performed to evaluate the associations between individual variables.

Results: ROP was observed in 29% of enrolled neonates, with significant associations identified between ROP and gestational age (p = 0.03), birth weight (p = 0.01), maternal gestational diabetes mellitus (GDM) (p = 0.03), neonatal apnea (p = 0.02), and oxygen therapy (p = 0.03). These findings highlight the substantial burden of ROP and its association with modifiable and non-modifiable factors. Additionally, the study suggests a potential emerging role of GDM as a risk factor for ROP.

## Introduction

Retinopathy of Prematurity (ROP) is a vasoproliferative retinal disorder primarily affecting premature and low birth weight neonates [1], often associated with prolonged oxygen exposure. It progresses in two phases: initial suppression of retinal vessel growth due to hyperoxia, followed by abnormal neovascularization driven by hypoxia [2]. The incidence of ROP in India is approximately 38%, and it has emerged as a leading cause of preventable blindness in low- and middle-income countries [3]. According to the International Classification of Retinopathy of Prematurity (ICROP), ROP is staged based on ophthalmoscopic findings at the junction of the vascularized and avascular retina, ranging from a faint demarcation line (stage 1) to complete retinal detachment (stage 5), with intermediate stages including an elevated ridge (stage 2), extraretinal fibrovascular proliferation (stage 3), and subtotal retinal detachment (stage 4). The presence of “plus disease“, characterized by dilation and tortuosity of the posterior retinal vessels, indicates increased disease severity [4].

Improved neonatal care has increased the survival of extremely premature infants, expanding the threshold of viability but also increasing the population at risk for complications such as ROP. As the neonatal mortality rate declined from 52 per 1,000 live births in 1990 to 28 per 1,000 live births in 2013, the incidence of ROP has risen proportionately. Rising preterm birth rates, improved survival, variability in care, and delayed diagnosis have collectively contributed to the growing burden of ROP-related blindness worldwide. Early diagnosis and appropriate intervention using laser photocoagulation, anti-vascular endothelial growth factor (anti-VEGF) therapy, or cryotherapy can substantially improve visual outcomes. Consequently, identifying both neonatal and maternal risk factors associated with ROP has become essential for improving screening strategies and prevention. In addition to well-established neonatal factors, limited evidence exists regarding maternal conditions such as gestational diabetes mellitus (GDM), which may influence retinal vascular development through metabolic and inflammatory pathways.

The objectives of this study were to determine the incidence of ROP among preterm and low birth weight neonates admitted to a tertiary care neonatal intensive care unit (NICU); to identify maternal risk factors associated with ROP; to evaluate neonatal risk factors related to ROP; and to assess the stage, zone, and presence of plus disease in affected neonates and correlate these findings with the identified risk factors.

## Materials and methods

Study design and sample size calculation

A single-center, retrospective observational study was conducted among neonates admitted to the neonatal intensive care unit (NICU) of a tertiary care hospital in Telangana, India, between December 1, 2023, and January 31, 2024. The study duration of two months was selected in accordance with resource limitations, investigator availability, and the Indian Council of Medical Research (ICMR) Short-Term Studentship (STS) guidelines, which specify a two-month research period. Despite the limited duration, the study yielded an adequate sample size to evaluate associations between risk factors and ROP.

The total sample size was calculated according to the formula: N=z²pq/L² (where z=1.96, p stands for prevalence (38.6%), q = 100 - p and L = absolute error of 10%) [5]. Based on the calculation, the required sample size was n = 100.

Study population

The study participants were selected per the following inclusion and exclusion criteria. Inclusion criteria: preterm neonates with gestational age <37 weeks and/or neonates with birth weight <2500 g. Exclusion criteria: neonates with major congenital abnormalities, neonates with ocular conditions preventing adequate fundus visualization, parental refusal to participate, and incomplete clinical documentation.

Data collection

Maternal and neonatal data were extracted from maternal and neonatal case records into a structured case report form. Maternal variables included gestational diabetes mellitus, gestational hypertension, pre-eclampsia with severe features, eclampsia, hypothyroidism, multiple gestation, mode of delivery (normal vaginal delivery or cesarean section), antenatal steroids administration, and premature rupture of membranes.

Neonatal variables included cardiopulmonary resuscitation, intubation, sepsis, apnea, respiratory distress syndrome, blood transfusions, oxygen therapy, neonatal jaundice, phototherapy for jaundice, intraventricular hemorrhage, surfactant administration, and laboratory parameters such as hemoglobin concentration, red blood cell count, platelet count, C-reactive protein (CRP), and blood culture results. Neonatal sepsis was diagnosed based on clinical features supported by laboratory findings, including a positive blood culture, elevated CRP levels, and leukocyte count abnormalities.

Ophthalmological examination

ROP screening was performed in the neonatal intensive care unit under aseptic precautions, with pupil dilation achieved using 1% tropicamide and 5% phenylephrine hydrochloride. Retinal examination was conducted by a qualified ophthalmologist using an indirect ophthalmoscope, aided by a wire eyelid speculum (L=44 mm, l=30 mm, H=12 mm, and lid blades of 6 mm and 9 mm) and loop depressor (125 mm length and 6.2 mm diameter) when necessary. A +28 diopter convex lens was also used when necessary. ROP findings were classified according to the ICROP Third Edition 2021, based on stage, zone, presence or absence of plus/pre-plus disease, and extent of involvement. All examinations were performed by a single ophthalmologist to maintain consistency.

Statistical analysis

Statistical analysis was performed using SPSS software, version 23 (IBM Corp., Armonk, NY). Univariate analysis was performed to identify significant risk factors, Student's t-test for continuous variables with normal distribution, Mann-Whitney U-test for variables with skewed distribution, and Chi-square test for categorical variables. A p-value less than 0.05 was considered statistically significant.

## Results

One hundred neonates who satisfied the inclusion criteria were included in the study. There were 53 (53%) males and 47(47%) females. The mean gestational age was 32.88 ± 3.00 weeks, and the mean birth weight was 1642 ± 533.26 g. The mean maternal age at delivery was 25.38 years ± 4.09. ROP was observed in 29 neonates, representing 29%. Seventy-one neonates had no ROP (71%).

Ophthalmologic findings

In the left eye, zone involvement included Zone 1 in eight neonates, Zone 2 in 32 neonates, and Zone 3 in 60 neonates. Stages 1, 2, 3, and 5 were observed in five (50%), three (30%), one (10%), and one (10%) neonate, respectively. Plus disease was present in 15 neonates, while 85 showed no signs of it. In the right eye, zone involvement included Zone 1 in eight, Zone 2 in 33, and Zone 3 in 59 neonates. Stages 1, 2, 3, and 5 were seen in four (50%), two (25%), one (13%), and one (13%) neonate, respectively, with no Stage 4 cases. Plus disease was present in 17 neonates, while 83 showed no signs of it. The association between neonatal gender and ROP was assessed using the Chi-square test. Among males, 15 had ROP, and 38 did not; among females, 14 had ROP and 33 did not. There was no significant association between gender and ROP (χ²(1) = 0.03, p = 0.86)

Maternal factors

The mean maternal age was 24.82 ± 3.91 years for neonates with ROP and 25.60 ± 4.17 years for those without ROP, with no statistically significant difference, t(98) = −0.88, p = 0.38. Neonates with ROP had a lower mean gestational age compared to those without ROP (31.96 ± 3.08 weeks vs 33.25 ± 2.90 weeks). A significantly greater proportion of ROP cases occurred among neonates born before 32 weeks (48.3% vs 22.5%) (Table 1).

**Table 1:** Association between gestational age and ROP *A statistically significant association was identified using Chi-Square test (χ²(2) = 6.83, p = 0.033). ROP: Retinopathy of prematurity.

Gestational Age	With ROP	Without ROP	p value
Extremely preterm (<28 weeks)	02 (6.9%)	02 (2.8%)	0.03*
Very preterm (28 to <32 weeks)	12 (41.4%)	14 (19.7%)	
Moderate to late preterm (32 to 37 weeks)	15 (51.7%)	55 (77.5%)	

Among neonates with ROP, 24.1% had mothers with GDM compared to 8.4% without ROP, showing a significant association (p = 0.03). No significant associations were found with other maternal parameters (Table 2).

**Table 2:** Association between maternal parameters and ROP ROP: Retinopathy of prematurity, PROM: Premature rupture of membranes. *The association was statistically significant based on the Chi-square test (χ²(1) = 4.63, p = 0.031).

Parameter	With ROP	Without ROP	p value
Gestational Diabetes mellitus			
Mothers with GDM	07 (24.1%)	06 (8.4%)	0.03*
Mothers without GDM	22 (75.9%)	65 (91.6%)	
Hypertension			
Yes	14 (48.3%)	23 (32.4%)	0.1
No	15 (51.7%)	48 (67.6%)	
Severe Pre-Eclampsia			
Yes	04 (13.8%)	08 (11.3%)	0.7
No	25 (86.2%)	63 (88.7%)	
Eclampsia			
Yes	05 (17.2%)	06 (8.5%)	0.2
No	24 (82.8%)	65 (91.5%)	
Hypothyroidism			
Yes	09 (31.1%)	14 (19.7%)	0.2
No	20 (68.9%)	57 (80.3%)	
Gestation			
Single	27 (93.1%)	50 (70.4%)	0.5
Twin	02 (6.9%)	21 (29.6%)	
Mode of delivery			
Normal vaginal delivery	16 (55.2%)	34 (47.9%)	0.1
Caesarean section	08 (27.6%)	32 (45.1%)	
Instrumental vaginal delivery	05 (17.2%)	05 (7%)	
Antenatal steroid therapy			
Yes	12 (41.4%)	20 (28.2%)	0.1
No	17 (58.6%)	51 (71.8%)	
PROM			
Yes	08 (27.6%)	13 (18.3%)	0.3
No	21 (72.4%)	58 (81.7%)	

Neonatal factors

Neonates with ROP had a mean birth weight of 1480.34 ± 463.08 g, compared to 1708.73 ± 548.69 g in those without ROP. A significantly higher proportion of ROP cases had birth weight <1500 g (58.6% vs 28.2%, p = 0.01) (Table 3).

**Table 3:** Association between birth weight and ROP *The association was statistically significant based on Chi-square test (χ²(2) = 8.20, p = 0.017).

Birth weight	With ROP	Without ROP	p value
Low Birth weight (<2500 to >1500 gm)	12 (41.4%)	51 (71.8%)	0.01*
Very low birth weight (<1500 to 1000 gm)	16 (55.2%)	19 (26.8%)	
Extremely low birth weight (<1000 gm)	01 (3.4%)	01 (1.4%)	

Among neonatal parameters, apnea showed a significant association with ROP (χ²(1) = 5.72, p = 0.02). Neonatal sepsis was defined based on clinical features supported by positive blood culture results. No significant associations were observed between ROP and early-onset sepsis, late-onset sepsis, cardiopulmonary resuscitation, 1-minute APGAR score, or the need for intubation. The 5-minute APGAR score showed a trend toward statistical significance (Table 4).

**Table 4:** Association between neonatal parameters and ROP APGAR: Appearance, Pulse, Grimace, Activity, and Respiration, CPR: Cardiopulmonary resuscitation.

Parameter	With ROP	Without ROP	p value
APGAR at 1 min (Mean±SD)			
	5.79±1.20	6.07±1.25	0.3
APGAR at 5 min (Mean±SD)			
	7.65±1.36	8.05±1.02	0.1
CPR			
Yes	4 (13.8%)	10 (14.1%)	0.9
No	25 (86.2%)	61 (85.9%)	
Intubation			
Yes	6 (20.7%)	9 (12.7%)	0.3
No	23 (79.3%)	62 (87.3%)	
Early Onset Sepsis			
Yes	16 (55.2%)	40 (56.3%)	0.9
No	13 (44.8%)	31 (43.7%)	
Late Onset Sepsis			
Yes	1 (3.4%)	3 (4.2%)	0.8
No	28 (96.6%)	68 (95.8%)	

There was no significant difference in hematological parameters between neonates with and without ROP. Mean hemoglobin levels were 14.12 g/dL vs. 13.85 g/dL (t(98) = 0.32, p = 0.75), RBC counts were 4.13 vs. 4.26 million/mm³ (t(98) = −0.48, p = 0.63), and platelet counts were 148.48 vs. 186.8 thousand/µL (t(98) = −1.36, p = 0.18), respectively. Oxygen therapy showed a significant association with ROP, with 65.5% of affected neonates receiving oxygen compared with 42.3% of those without ROP. However, the mean oxygen flow rate did not significantly differ between groups. Respiratory support parameters showed no significant association with ROP. Use of Bubble continuous positive airway pressure (BCPAP), maximum fractional inspired oxygen (FiO₂), minimum FiO₂, and positive end expiratory pressure (PEEP) levels were not significantly associated with ROP (Table 5).

**Table 5:** Association between neonatal parameters and ROP *A statistically significant association was identified using the Chi-square test (χ²(1) = 4.74, p = 0.029). BCPAP: Bubble continuous positive airway pressure, ROP: Retinopathy of prematurity.

Parameter	With ROP	Without ROP	p value
Oxygen			
Received oxygen therapy	19 (65.5%)	30 (42.3%)	0.03*
No oxygen therapy	10 (34.5%)	41 (57.7%)	
O₂ flow (L/min)			
Mean ± SD	1.17 ± 1.44	1.74 ± 2.50	0.2
Bubble CPAP (BCPAP)			
Yes	11 (37.9%)	24 (33.8%)	0.7
No	18 (62.1%)	47 (66.2%)	
Maximum FiO₂			
Mean ± SD	23.62 ± 37.24	21.84 ± 35.38	0.8
Minimum FiO₂			
Mean ± SD	3.10 ± 11.68	2.39 ± 12.47	0.7
Positive end-expiratory pressure (PEEP)			
Mean ± SD	1.62 ± 2.47	1.29 ± 2.25	0.5

Further analysis of neonatal factors showed no significant associations between ROP and neonatal jaundice (NNJ), phototherapy, surfactant use, thrombocytopenia, necrotizing enterocolitis (NEC), or early breastfeeding. However, intraventricular hemorrhage (IVH) showed a trend toward significance (p = 0.1), suggesting a potential association warranting further study.

## Discussion

ROP remains a leading cause of preventable blindness worldwide and a significant morbidity in premature infants, with incidence increasing as gestational age and birth weight decrease [6]. In our study, 29% of neonates developed ROP, which is consistent with previous reported Indian rates ranging from 21.6% and 46% [7] and is comparable to the 27% incidence reported by Maheshwari et al. [8]. Higher incidences have been reported in the Middle East, including 56% in Saudi Arabia and 37.4% in a NICU in Riyadh [9]. These variations reflect differences in screening criteria, gestational age, and birth weight thresholds used across studies.

Among neonates with ROP in our study, Stage 1 disease was the most common. accounting for 50% cases, while the remaining cases included Stages 2, 4, and 5, with one neonate presenting with Stage 5 ROP and total retinal detachment [10]. Our findings are consistent with the Swiss Society of Neonatology’s stage-wise distribution of ROP. Plus disease, a predictor of disease progression, was observed in 15% of left eyes and 17% of right eyes. Neonates with plus disease are more likely to progress to retinal detachment compared with those without plus disease [8]. Gender was not significantly associated with ROP incidence in our study (p = 0.8), consistent with findings from South India but contrasting with a meta-analysis that reported male sex as a risk factor for severe ROP (relative risk (RR) = 1.14, CI: 1.07-1.22) and a lower risk of non-severe ROP (RR = 0.92, 95% CI: 0.87-0.97) [11].

Birth weight showed a significant association with ROP, with 55.6% of neonates with ROP having a birth weight <1500 grams, compared to only 30% of neonates without ROP. Although most cases occur in very low birth weight infants, more than 40% of affected neonates in our study weighed between 1500 and 2500 grams, underscoring the need for broader screening criteria. The mean birth weight of babies with ROP in our study was 1480 grams, slightly higher than the mean birth weights of 1344 grams (in urban NICUs) and 1375 grams (in semiurban NICUs) as reported by Murthy et al. [12]. Lower birth weight showed a significant association with ROP, with very low and extremely low birth weight neonates at higher risk. Low birth weight and small for gestational age increase the likelihood of requiring ROP treatment [13]. In a study by Ahuja et al., low birth weight was found to be a significant and independent risk factor for ROP [14]. A potential reason for the increased prevalence of ROP among infants with low birth weight could be attributed to the findings indicating a significantly larger nonvascularized area in the temporal retina. Consequently, the expanded avascular retina might serve as a more substantial hypoxic trigger for the subsequent release of angiogenic factors, leading to the onset of severe ROP.

Gestational age also had a significant association with ROP, with 48.3% of neonates with ROP being <32 weeks compared to 22.5% of those without. These findings are consistent with Murthy et al., who reported a higher prevalence of ROP in neonates with lower gestational ages [12]. The mean gestational age of neonates with ROP in our study was 32 weeks, slightly higher than that reported in studies from other countries. For example, a study done by Shah et al. [16] showed that the mean gestational age in the cohort with ROP was 29.7 (SD = 2.2) weeks, and Globe et al. [15] reported a mean gestational age of 29.1 (SD-2.1) weeks, potentially reflecting higher mortality rates in extremely preterm neonates in our setting.

Oxygen therapy was significantly associated with ROP, with 65.5% of affected neonates receiving oxygen compared with 42.3% without ROP. However, the oxygen flow rate did not show a significant correlation. Oxygen impacts retinal vasculature in two phases: an initial vasoconstrictive phase suppressing VEGF, followed by a vasoproliferative phase triggered by hypoxia-induced VEGF overproduction [16]. This dual-edged nature of oxygen therapy necessitates careful monitoring to balance risks of hypoxia and hyperoxia, wherein hypoxia poses elevated risks of mortality, cerebral palsy, patent ductus arteriosus, pulmonary hypertension and apnea, while on the other hand, hyperoxia is associated with higher rates of morbidity, including diseases such as retinopathy of prematurity and bronchopulmonary dysplasia (BPD), etc.

A study by Suga et al. reported that CPAP use was protective against developing ROP requiring treatment [17]. However, our study did not find any significant associations between ROP and CPAP use, mean maximum/minimum FiO2, or the mean positive end expiratory pressure (PEEP) used. Apnea was another significant risk factor, with over one-third of neonates with ROP having a history of apnea compared to 14% without ROP. Our results on apnea coincide with those of Gaber et al. [18] and also with the results of Suga et al. [17]. Oxygen fluctuations in the first 30 days of life increase ROP risk, explaining the higher prevalence in infants with apnea [19]. Conversely, maternal age showed no significant association with ROP in our study, although recent research suggests advanced maternal age may contribute to preterm birth and low birth weight, both established ROP risk factors [20]. This could be attributed to the rising average age of marriage, resulting in an increasing average maternal age at childbirth. Moreover, with increasing maternal age, the risk of low birth weight and preterm delivery increases. Both of these are associated with the risk of ROP. Since our study was based in a government tertiary care center where most mothers are generally young, as they come from rural and underdeveloped areas, maternal age did not show any significant association.

GDM was the only maternal factor significantly associated with ROP, with nearly 25% of affected neonates born to mothers with GDM compared to 10% in the non-ROP group. A few other studies by Tunay et al. [21] and Opara et al. [22] have also iterated that GDM increases the risk of ROP in the neonates. Other maternal factors, such as hypertension, pre-eclampsia, multiple gestation, and delivery mode, did not show significant associations, aligning with findings from Hungi et al. [23]. Antenatal steroid administration also had no protective effect against ROP.

While some studies link lower APGAR scores to ROP, multivariate analysis often finds no significant association, as in our study [24]. Similarly, CPR showed no correlation with ROP, contradicting most studies. The association in other research may stem from underlying systemic illness, hypoxic episodes, or post-CPR hyperoxia affecting retinal vasculature [25]. Contrary to studies such as that by Sugo et al. [17], our findings did not support significant associations between ROP and conditions such as sepsis, intubation, blood transfusions, or respiratory distress syndrome (RDS). Likewise, our study did not show any significant association between surfactant therapy and ROP. Studying the correlation between anemia and ROP is complex due to multiple factors affecting hemoglobin and hematocrit levels, notably blood transfusions [26]. In our analysis, hemoglobin, RBC count, and platelet levels did not significantly differ between neonates with and without ROP. This contrasts with previous studies suggesting thrombocytopenia may delay normal retinal vascularization and promote uncontrolled neovascularization due to reduced levels of VEGF, IGF-1, and PDGF [27].

Phototherapy and neonatal jaundice, previously suggested as protective against ROP, showed no significant association in our study. Additionally, breastfeeding, which has been proposed as protective against ROP in a study by Manzoni et al. [28], did not show a significant correlation (p = 0.4) in our study. Likewise, surgical necrotizing enterocolitis (NEC) was not significantly associated with ROP, contradicting some prior studies [29]. It is essential to balance the advantages of identifying as many cases of ROP as feasible with the drawbacks of excessive screening, such as discomfort for infants, increased expenses, and the scarcity of adequately trained clinicians to conduct the standard screening examinations [30]. Recent studies have suggested adopting a risk factor-oriented strategy for ROP screening in infants who do not meet conventional screening criteria, especially in underdeveloped, low- and middle-income regions.

## Conclusions

Our study identified several neonatal, hospitalization, and maternal risk factors associated with ROP and highlights the emerging role of maternal GDM as a potential contributor to ROP development. These findings emphasize the need for tailored screening guidelines specific to our hospital setting to improve early detection and resource allocation. While our findings align with many previous studies, the lack of significant associations for some expected risk factors may be attributable to the modest sample size (n = 100) and the tertiary-care referral nature of the NICU, which may influence case distribution.

Future research should explore these associations in larger, multicenter cohorts, including primary and secondary care units, to improve generalizability. Longitudinal studies on ROP progression and treatment response could further refine screening and management strategies, ultimately improving outcomes for premature infants at risk of vision impairment.
